# The regulatory roles of Smad2/3 protein and *SMURF2* gene expression in granulosa cells of germinal vesicle and metaphase II oocytes in polycystic ovarian syndrome: A case-control study

**DOI:** 10.18502/ijrm.v22i6.16794

**Published:** 2024-08-05

**Authors:** Marzieh Ghorbani, Marzieh Sanoee Farimani, Iraj Khodadadi, Sina Mohagheghi, Iraj Amiri, Heidar Tayebinia

**Affiliations:** ^1^Department of Clinical Biochemistry, School of Medicine, Hamadan University of Medical Sciences, Hamadan, Iran.; ^2^Fertility and Infertility Research Centre, Hamadan University of Medical Sciences, Hamadan, Iran.; ^3^Department of Obstetrics and Gynecology, Medicine School, Hamadan University of Medical Sciences, Hamadan, Iran.; ^4^Omid Infertility Centre, Hamadan, Iran.; ^5^Department of Anatomy and Embryology, School of Medicine, Hamadan University of Medical Sciences, Hamadan, Iran.

**Keywords:** Polycystic ovary, Oocytes, Granulosa cell.

## Abstract

**Background:**

The impaired functions of granulosa cells (GCs) in the delayed development and immaturity of oocytes have been reported in polycystic ovary syndrome (PCOs). Even with ovarian stimulation, a large number of oocytes in these patients are still in the stage germinal vesicle (GV).

**Objective:**

The levels of Smad2/3, phosphorylated Smad2/3 (P-Smad2/3), the expression of *SARA*, *Smad4*, and *SMURF2 *genes in the GCs surrounding metaphase II (MII) or GV oocytes in PCOs women were investigated.

**Materials and Methods:**

GCs of MII and GV oocytes were isolated from 38 women with PCOs and the expression levels of *SARA, Smad4,* and *SMURF2 *in surrounding GCs of MII and GV oocytes were determined using reverse-transcription polymerase chain reaction*.* Also, Smad2/3 and P-Smad2/3 proteins were determined using western blotting.

**Results:**

The expression level of *SMURF2* was significantly higher in GCs surrounding GV oocytes compared with that of GCs encompassing MII oocytes (p < 0.001). At the same time, no significant differences were observed in *SARA* and *Smad4* expression levels in GCs surrounding GV and MII oocytes. A lower level of P-Smad2/3 was also found in GCs GV oocytes compared with GCs of MII oocytes (p < 0.001).

**Conclusion:**

It seems that P-Smad2/3 plays a role in oocyte development, and the downregulation of this protein is associated with a defect in the maturation of GV oocytes. On the other hand, the upregulation of the *SMURF2* gene also affects the growth process of GCs and the maturation of GV oocytes.

## 1. Introduction

Polycystic ovary syndrome (PCOS) as an endocrine disorder is seen in 8–13% of women of reproductive age. It manifests critical features, including increased androgen levels, multiple small cysts, dysregulated menstrual cycles, hirsutism, and anovulation (1, 2), together with the presence of poor-quality oocytes and infertility in 74% of these women.

The process of folliculogenesis is disturbed in PCOS, and in these women, the primary follicles stop in the early preantral stages. Women with PCOS need assisted reproductive techniques, and high-dose gonadotropins are prescribed for controlled ovarian hyperstimulation, resulting in abundant follicles with poor quality, unequal size, and maturation spectrum, immature oocytes-germinal vesicles (GV) and mature oocytes-meiosis II (3–5).

In the folliculogenesis pathway of the mammalian ovary, there is a precise biological interaction between oocytes, granulosa cells (GCs), cumulus cells, and theca cells (TCs). Investigations found that the formation of immature oocytes could be aroused from insufficient interaction between these cells. Therefore, understanding signaling pathways in ovarian development is essential for increasing the maturation rate of oocytes and managing infertility. Factors produced in the ovary such as growth differentiation factor 9 (GDF9) and bone morphogenetic protein 15, also known as GDF9b, and anti-Müllerian hormone (AMH) as proteins of the transforming growth factor β (TGF β ) superfamily are regulators of the follicular environment and determine the fate of each follicle (6). Studies have shown that in the absence of GDF9, the TCs layer is not formed, the production of steroids decreases, and the follicles remain immature (7, 8).

One of the signaling pathways is the TGF β /mothers against decapentaplegic homolog (Smads) pathway, which plays a role in the regulation of follicular growth, and disruption of this pathway inhibits follicular growth and ultimately results in follicular atresia (9). TGF β phosphorylates the kinase domain of type I receptors. Phosphorylated receptors transmit the signal through the phosphorylation of proteins called Smads. Smad proteins are classified into 3 subclasses based on their intracellular functions: R-Smads (Smad1, 2, 3, 5, and 8), Co-Smads (Smad4), and I- Smads (Smad6 and 7). Phosphorylated Smad2/3 (P-Smad2/3) with the cooperation of Smad4 complexes, transmit signals to the nucleus and regulate the transcription of target genes (6). These proteins play an essential role in all stages of follicular development (10). Also, Smad anchors for receptor activation (*SARA*) control and facilitates the localization of Smad 2/3 to be presented to the receptors (11). Furthermore, Smad ubiquitination regulatory factor 2 (*SMURF2*) is one of the proteins belonging to E3 ubiquitin ligase and is known as a negative regulator in this signaling pathway (12).

Women with PCOS often have poor oocyte quality and maturation, which affects their fertility and response to assisted reproductive techniques and ovulation induction drugs. The average development and growth of the follicles depends on the complex interaction between the oocytes and the surrounding GCs, which are influenced by signaling pathways such as Smad. Relative concentrations were compared with beta-actin protein and calculated by ImageJ-1.52 software.

In this study, the levels of Smad2/3 and P-Smad2/3 proteins and the expression of *SARA*, *Smad4*, and *SMURF2* genes were investigated in the GCs of GV and MII oocytes in women with PCOS. By comparing changes in the expression levels of these molecules in the GCs of GV and MII oocytes, we hope to gain more insight into the molecular mechanisms underlying the impaired oocyte maturation in women with PCOS.

## 2. Materials and Methods 

### Study population-study design

This case-control study was conducted among 38 PCOS women aged between 25 and 38 yr who were admitted to Omid Clinic (Hamadan, Iran) as in vitro fertilization procedure candidates from August 2021-February 2022. PCOS was diagnosed based on the Rotterdam criteria (13), and all of them were under treatment with ovulation induction drugs. The exclusion criteria were the presence of hyperprolactinemia, congenital adrenal hyperplasia, and Cushing syndrome. Follicle-stimulating hormone (FSH), luteinizing hormone, estradiol, AMH, prolactin, and testosterone were evaluated in their serum at the start of the controlled ovarian stimulation (1–3 days of menstruation).

### Sample size

Considering that a similar study has not been conducted in humans in relation to the objectives of this study, a pilot study was conducted. Based on the study findings, the mean and standard deviation in the mature and immature cells of the participants were calculated considering the power of the test equal to 0.9 and the type 1 error equal to 0.05. Then using the following statistical formula, the average variances of gene expression in 2 groups of mature and immature cells, the initial sample size was estimated at 25, and 38 people were considered people in each group to increase the power of statistical tests.

n= (Z ${\;}_{1-\alpha /2}$ + Z ${\;}_{1-\beta }$ ) ${\;}^{2}$ (Z ${\;}_{1}{\;}^{2}$ + Z ${\;}_{2}{\;}^{2}$ )/(µ ${\;}_{1}$ - µ ${\;}_{1}$ ) ${\;}^{2}$

### Ovarian stimulation process

The ovarian stimulation process was carried out according to antagonist protocols, recombinant FSH and Cinnal-FⓇ (CinnaGen Co., Tehran, Iran) were administered. When the dominant follicle reached a size of 14 mm, a Cetrotide antagonist (Merck, Darmstadt, Germany) was injected at a daily dose of 0.25 μ g. Once at least 3 mature follicles (18 mm) were observed in the ovaries, recombinant Ovitrelle-hCG (Merck, Darmstadt, Germany) was administered. After a 36-hr interval, follicles were obtained via transvaginal ultrasound-guided puncture, and oocytes were isolated.

### Isolation of GCs

Following the retrieval of oocytes, a complete denudation and maturity assessment process was carried out. The identification of MII oocytes was based on the first polar body's presence, while the GVs presence identified GV oocytes. The average MII oocyte number of women was 17.17 ± 1.34 whereas 5 ± 2 GV oocytes were retrieved from each subject. Then, GCs surrounding MII oocytes or GVs were separately collected and designated as GC-MII and GC-GV groups, respectively. The collected cells were carefully transferred into micro tubes and centrifuged before being analyzed in quantitative real-time polymerase chain reaction (RT-qPCR) and Western blotting techniques.

### Identification of GCs 

**Figure 1:**
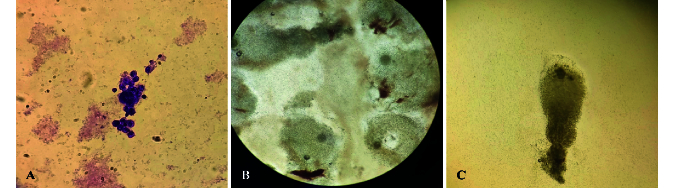
Granulosa cells and mature and immature oocytes. A) Granulosa cells with Wright-Giemsa staining. B) MII oocytes with expanded distal layers of GCs. C) GV oocytes with compacted proximal layers.

Following isolating GCs (0.1–0.6 × 10^6^ cells per each oocyte), Wright-Giemsa staining was used (Figure 1).

### RT-qPCR for gene expression assay

After designing the primers, the sequence of each primer was checked and reconfirmed with Primer Blast software to evaluate the specificity to the corresponding gene. Total RNAs were extracted from GCs using RNX PLUS (SinaClon, Iran). The concentration of extracted RNA was evaluated using a NanoDrop ${\;}^{\text{TM}}$ spectrophotometer (Thermo Fisher Scientific-USA) and its quality was evaluated by 1% agarose gel electrophoresis. The concentration of all total RNAs was adjusted to 500 ng/mL and reversely transcribed into cDNA using a cDNA synthesizing kit (Parstous Biotechnology, Iran). RT-qPCRwas performed using RealQ Plus Master Mix Green (Sina SYBR Blue NO ROX HS-qPCR Kit) in a Roche LightCyclerVR96 System (Roche Life Science, Sandhofer, Germany). The relative expression of *SARA*, *Smad4* and *SMURF2* genes was calculated using the 2 ${\;}^{-\Delta \Delta Ct}$ method. Act B (Actin Beta) was used as a reference gene. All samples were prepared in duplicate, and the mean value was used for comparative analyses (Table I).

### Western blotting for the protein assay

Western blotting for detection of Smad2/3 and P-Smad2/3 was performed. A mixture of 450 μ l Radioimmunoprecipitation assay buffer (Cytomatingene, Iran) and 6 μ l of protease inhibitor (Cytomatingene, Iran) was used to lyse 1 × 10^6^ GCs. The cell lysates were centrifuged at 12,000 × g for 20 min. The cell-soluble proteins were collected from the supernatants and transferred to new microtubes. Then, the total protein contents were determined using a Bicinchoninic Acid Protein Assay kit (DNAbiotech, Iran), where bovine serum albumin was used as standard. For western blotting, protein electrophoresis was performed using sodium dodecyl sulfate-polyacrylamide gel (SDS-PAGE) and then protein bands were transferred to polyvinylidene fluoride membrane. For blocking, 5% non-fat milk in Tris-buffered saline tween was added for 2 hr on polyvinyl chloride membrane, then primary antibodies against P-Smad2/3 (1:2,000 ab63399), Smad2/3 (1:2,000 ab63672), and β -actin (1:2,000 ab8227) were added for 12 hr. Membranes were washed 3 times with TBST buffer, then incubated with secondary antibody (Anti-rabbit IgG, HRP-Linked Antibody 7074) and stained with enhanced chemiluminescence (Cytomatingene, Iran). The protein bands were captured on film in the dark. Finally, Image J-1.52 software was used to quantify protein expression levels relative to β -actin as the control.

**Table 1:** Nucleotide sequences of primers used for qRT-PCR amplification


**Gene**	**Primer strand**	**Sequence**
	Forward	CAGTGCTAATGAATGCTCAA
***SARA***	Reverse	GTGCTTTAAAACTCCCACAG
		Forward	GAGTGGGTTTCAGTCTGTGT
***SMURF2***	Reverse	CCCCTCTTCTCTTAAACCAT
		Forward	TAGGCAAGGAAACCAGATAA
***Smad4***	Reverse	AATGCCAATACAATGAGGTC
		Forward	AAGATCAAGATCATTGCT
***ActB***	Reverse	TAACGCAACTAAGTCATA
	qRT-PCR: Quantitative real-time polymerase chain reaction, *SARA*: Smad anchors for receptor activation, *SMURF2*: Smad ubiquitination regulatory factor 2, *Smad4*: Mothers against decapentaplegic homolog 4, ActB: Actin Beta		

### Ethical considerations

The study was approved by the Research Ethical Committee of Hamadan University of Medical Sciences, Hamadan, Iran (Code: IR.UMSHA.REC.1400.244). The main objectives of the study were explained to the women with PCOS, and written informed consents were signed. All study procedures were performed under the ethical guidelines of the Declaration of Helsinki (1967 version 2013) and with the ethical standards of the National Iranian Research Committee.

### Statistical analysis

The results were shown as mean ± SEM. The normal distribution of data was evaluated using the Kolmogorov-Smirnov test. Data were statistically analyzed using Student's *t* test for the data with normal distributions and Mann-Whitney tests were also applied for the data with abnormal distributions. Furthermore, results were analyzed using the GraphPad Prism 7.05 software (USA). Statistical significance was assumed at the p < 0.05. Statistical analysis was performed using the statistical package for social sciences logistic regression (SPSS, Inc., Chicago, IL) version 16.0. Relative concentrations were compared with beta-actin protein and calculated by ImageJ-1.52 software.

## 3. Results

### Demographic characteristics of women with PCOS

Table II shows that the studied women aged between 25 and 38 yr. It is of considerable importance to note that a comprehensive evaluation of the women's serum hormone levels was conducted before the start of the controlled ovarian stimulation. This evaluation included FSH, luteinizing hormone, estradiol, AMH, prolactin, Thyroid-stimulating hormone, and testosterone.

### The gene expression levels of *SARA*, *Smad4*, and *SMURF2*

**Figure 2:**
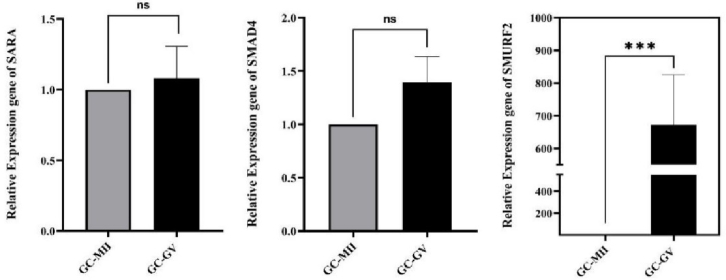
RT-PCR analysis of *SARA*, *Smad4, and SMURF2* in surrounding granulosa cells of immature oocytes (GV) compared to the surrounding granulosa cells of mature oocytes (MII) in women with PCOS (n = 38). RT-PCR results were normalized to beta-actin and are displayed as Mean ± SEM. ns: Not significant, ***P < 0.001.

RT-qPCR was applied to determine the gene expression levels of *SARA*, *Smad4*,and* SMURF2 *in GCs of GV and MII oocytes. As shown in figure 2, the *SARA* and *Smad4* expression levels did not significantly differ in GCs surrounding GV oocytes and MII oocytes (p > 0.05). However, the expression of *SMURF2* was found to be considerably upregulated (p < 0.001) in GCs of GVs and rapidly increased (a few-hundred folds) compared to GCs of MII oocytes (Figure 2).

### The protein expression levels of Smad2/3 and P-Smad2/3 

**Figure 3:**
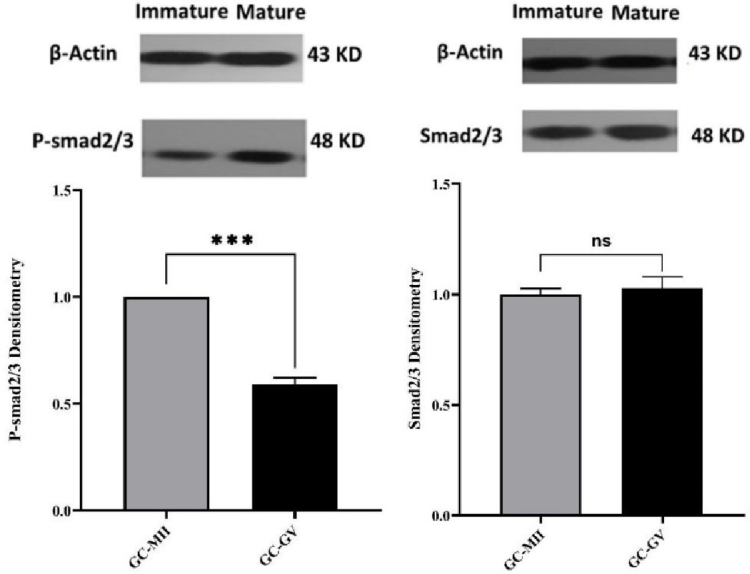
The P-Smad2/3 and Smad2/3 protein expression in granulosa cells surrounding immature oocytes (GV) compared to the surrounding granulosa cells of mature oocytes(MII) in women with PCOS (n = 38). The Western blotting bands intensity was quantified densitometrically and normalized to that of beta-actin, and are displayed as Mean ± SEM. ns: Not significant, ***P < 0.001.

The expression of Smad2/3 and P-Smad2/3 at the protein level was evaluated by Western blotting. No significant difference was observed in Smad2/3 protein levels between GCs surrounding MII and GV oocytes. In contrast, P-Smad2/3 was significantly lower (p < 0.001) in GCs surrounding GV compared with that of GCs from MII oocytes (Figure 3).

**Table 2:** Demographic characteristics and hormonal status in PCOS women


**Factors**	**PCOS (n = 38)**
**Duration of infertility (yr)**	6.48 ± 0.29
**BMI (kg/m^2^)**	29.10 ± 0.548
**No. of MII oocytes retrieved**	17.17 ± 1.34
**No. of GV oocytes retrieved**	5 ± 2
**FSH (IU/L)**	5.32 ± 0.429
**LH (IU/L)**	9.12 ± 0.360
**LH/FSH**	1.73 ± 0.313
**E2 (pg/ml)**	88.65 ± 2.23
**AMH (ng/ml)**	5.95 ± 0.396
**PRL (ng/ml)**	15.12 ± 1.62
**TSH ( μ U/ml)**	1.72 ± 1.23
**Testosterone (ng/ml)**	1.82 ± 0.121
Data presented as Mean ± SEM. PCOS: Polycystic ovary syndrome, BMI: Body mass index, MII: Metaphase II, GV: Germinal vesicle, LH: Luteinizing hormone, FSH: Follicle-stimulating hormone, E2: Estradiol, AMH: Anti-Müllerian hormone, PRL: Prolactin, TSH: Thyroid-stimulating hormone	

## 4. Discussion

Women with PCOS often have heterogeneous follicles, a high rate of immature GV oocytes, and inappropriate fertilization rates (14). Follicle growth depends on oocytes, GCs, and TCs. Communication between GCs and oocytes is essential for follicular development, because GCs provide a variety of factors for oocytes, and the function of GCs is often directly related to the quality of the oocytes (15). GCs are considered regulators of oocyte development and can serve as biomarkers for evaluating oocyte quality, embryo competence, and pregnancy outcome (16). Our study showed that the expression level of *SMURF2* was significantly higher in GCs surrounding GV oocytes compared with that of GCs encompassing MII oocytes, a lower level of P-Smad2/3 was also found in GCs of GV oocytes compared with GCs of MII oocytes. It was in line with our hypothesis that GCs of GV oocytes are different in terms of protein and gene expression.

Smads, as members of the TGF β family, play essential roles in the reproductive system, take part in multiple aspects of ovarian functions, and have great importance during oocyte maturation. They both stimulate FSH receptors and affect the stability of follicle-stimulating hormone receptor mRNA and in the absence of Smad, ovulation, and follicle growth are impaired. Smads also affect the function of FSH. Conversely, FSH can stimulate and activate Smad2/3 and Smad4. FSH controls ovarian follicular growth and leads to maturation and selection of the dominant follicle and estradiol production (17, 18).

In this study, the protein levels of Smad2/3 did not differ between MII and GV oocyte-surrounded GCs, whereas the P-Smad2/3 was significantly lower in the GV oocyte-surrounded GCs. The binding of TGF β to its receptor induces activation and phosphorylation of Smads 2 and 3. P-Smad2/3, with the cooperation of Smad4 complexes, transmit signals to the nucleus and regulate the transcription of target genes (19). It seems that, due to the decrease of P-Smad2/3 in GV oocyte-surrounded GCs, the effect of FSH had decreased and the ability of follicular growth and multiplication has reduced. Also, activation of Smad2/3 signaling machinery within the ovary mediates signals produced by the oocytes and is crucial for coordinating essential events of the ovulatory process, including granulosa expansion (20). GV oocyte-surrounded GCs did not have granulosa expansion, and their GCs remained compressed, while MII oocytes usually have a good expansion, which indicates their development (Figure 1).

It is reported that FSH reduces the apoptosis of GCs by decreasing FasL levels in vitro and protects the follicles from atresia in vivo (21, 22), and it prevents atresia of various maturing follicles, including antral follicles, pre-ovulatory follicles, and dominant follicles (23, 24). In addition, aromatase, an estradiol-producing enzyme, is stimulated by FSH in GCs, and the production of estradiol is critical for the selection of dominant follicles and follicular development (25, 26). We did not investigate the apoptosis of GCs, but it can be inferred that due to the reduction of P-Smad2/3, the function of FSH is impaired, and the apoptosis of GCs is not prevented, and these cells are stopped in the GV stages.

It is reported that the activation of Smad2/3 through phosphorylation is carried out via the TGF β receptor type I and the adapter molecule *SARA*, which facilitates the presentation of Smad2/3 to the TGF β 1 receptor (19). Then Smads 2/3 phosphorylated with Smad4 translocated to the nucleus and regulated the transcription of target genes (27). During oocyte development, the Smad4 protein and mRNA expression patterns play a role in folliculogenesis (28, 29).

Our results revealed that the expression levels of *SARA* and *Smad4* were not significantly different between the GCs surrounding MII and GV oocytes. And it can also be concluded that these genes do not have a significant effect on the proliferation and development of GCs. However, the expression of *SMURF2* belonging to E3 ubiquitin ligase and a negative regulator of TGF β signaling pathways was significantly upregulated in the GCs surrounding GV oocytes, with an approximately 672-fold increase. It is revealed that *SMURF2* suppresses the TGF β signaling pathway (30). Considering that our study examined both the gene expression and protein levels, in GCs, it was found that P-Smad2/3, the functional form of this protein, contains low expression proteins in GCs surrounding GV oocytes, it does not transmit signals to the nucleus to a sufficient extent and neither does it regulate the transcription of target genes.

Therefore, the role of P-Smad2/3 protein as stimulator FSH receptors, and stabilizer FSHR mRNA is not done correctly, and follicular development, GCs expansion, and maturation of GV oocytes are disturbed, causing the oocytes to remain immature (GV). On the other hand, the *SMURF2* gene is more expressed in GCs surrounding GV oocytes, which induces Smad2/3 degradation, and maybe this inhibitory factor restricts the development of the oocytes (25).

## 5. Conclusion

In summary, it seems that P-Smad2/3 plays an essential role in oocyte development, and the downregulation of this protein is associated with a defect in the maturation of GV oocytes. On the other hand, the upregulation of the *SMURF2* gene also affects the growth process of these cells with its inhibitory effect. Additional research is necessary to reveal the activity of the Smad2/3 pathway. Due to the increase in the number of GV oocytes in women with PCOS, it is suggested that the addition of recombinant P-Smad2/3 proteins and inhibitor of *SMURF2* may be effective in increasing the number of MII oocytes and fertility outcomes in these women and even treatment of human infertility using immature oocytes collected from natural cycles.

##  Data availability

Data supporting the findings of this study are available upon reasonable request from the corresponding author.

##  Author contributions

HT, MGh, and IK designed the study and wrote the paper, MSF, IA, and MGh contributed to sample collection, MGh, SM, IK, and HT contributed to performing experiments and evaluating the results. All authors approved the final manuscript and take responsibility for the integrity of the data and the accuracy of the data analysis.

##  Conflict of Interest

The authors declare that there is no conflict of interest.
